# Trading contact tracing efficiency for finding patient zero

**DOI:** 10.1038/s41598-022-26892-7

**Published:** 2022-12-30

**Authors:** Marcin Waniek, Petter Holme, Katayoun Farrahi, Rémi Emonet, Manuel Cebrian, Talal Rahwan

**Affiliations:** 1 Computer Science, Science Division, New York University Abu Dhabi, Abu Dhabi, UAE; 2 Aalto University, Espoo, Finland; 3 Kobe University, Kobe, Japan; 4 University of Southampton, Southampton, UK; 5 CNRS, Laboratoire Hubert Curien UMR 5516, UJM-Saint-Etienne, Université de Lyon, Saint-Étienne, France; 6 Center for Humans and Machines, Max Planck Institute for Human Development, Berlin, Germany; 7 Statistics Department, Universidad Carlos III de Madrid, Madrid, Spain; 8 UC3M-Santander Big Data Institute, Universidad Carlos III de Madrid, Madrid, Spain

**Keywords:** Computational science, Infectious diseases

## Abstract

As the COVID-19 pandemic has demonstrated, identifying the origin of a pandemic remains a challenging task. The search for patient zero may benefit from the widely-used and well-established toolkit of contact tracing methods, although this possibility has not been explored to date. We fill this gap by investigating the prospect of performing the source detection task as part of the contact tracing process, i.e., the possibility of tuning the parameters of the process in order to pinpoint the origin of the infection. To this end, we perform simulations on temporal networks using a recent diffusion model that recreates the dynamics of the COVID-19 pandemic. We find that increasing the budget for contact tracing beyond a certain threshold can significantly improve the identification of infected individuals but has diminishing returns in terms of source detection. Moreover, disease variants of higher infectivity make it easier to find the source but harder to identify infected individuals. Finally, we unravel a seemingly-intrinsic trade-off between the use of contact tracing to either identify infected nodes or detect the source of infection. This trade-off suggests that focusing on the identification of patient zero may come at the expense of identifying infected individuals.

## Introduction

Determining the origin of the COVID-19 pandemic, and the majority of globally emergent pathogens, is a challenging task, despite large technical advances in genetic epidemiology. The methods based on this approach attempt to determine the exact source of the virus via sequencing the virus genome and tracing different viral lineages^1^. While such techniques can help chart the general evolution of the virus, they require collecting biological samples in numerous locations and subjecting them to laborious analyses.

Alternative and complementary ways of seeking the source of a viral infection come from social network analysis^2–5^. These methods, called source detection algorithms, are designed to identify the origin of an infection with much greater granularity than the sequencing-based alternative. However, to work properly, these methods require extensive amounts of information, including the structure of the social connections and the infection state of the individuals at various points in time, which are often unavailable. In addition to the inadequacy of existing methods, there is another major obstacle facing the source-detection endeavour: the availability of limited resources that may be better spent on disease containment instead.

One strategy that got widely adopted worldwide to help curb the pandemic is contact tracing^6^ the practice of identifying people who came in close contact with a positive case. For years, contact tracing was used for disease control, in particular when it comes to sexually transmitted diseases and newly appearing pathogens^7^, e.g., it was successfully used to curb the spread of smallpox and SARS^8^. The effectiveness of contact tracing was further accelerated by the use of digital technologies^9^, and in this form, it was employed to curb the spread of COVID-19^6,10,11^. Since it might be impossible to recover the actual network of contacts in which the spreading of the disease took place, one can use the communication network as a proxy for the purpose of contact tracing^12^.

While existing research on contact tracing was focused on using it to detect either current or future infections, we investigate the potential use of contact tracing to detect the source of infection, adding a new dimension to the utility of the contact tracing toolkit. To this end, we perform a wide variety of simulations on different kinds of temporal network structures, while using a spreading model of the COVID-19 disease^13^ to recreate the dynamics of the epidemic. It is worth noting that many existing source detection methods would benefit from the information gathered using a contact tracing process, e.g., in order to recreate the structure of the social network under consideration. What we are concerned with in this work is performing the source detection task, *as part* of the contact tracing process, and not as a prerequisite or an introduction to another source detection method. We attempt to answer the question of whether it is possible to pinpoint patient zero by tuning the parameters of the contact tracing process. If this method proves to be effective, it might help us determine the origins of future diseases without hindering the efforts to keep those diseases at bay. Our analysis indicates that, depending on the parameterization of the contact tracing process, it is possible to identify the source of the infection, i.e., the network node that represents patient zero. The majority of our findings focus on an inherent compromise between identifying the source of infection and identifying the nodes that are currently infected when these two tasks share the same budget. In other words, getting closer to patient zero may divert the resources from obtaining information that can be used to improve our chances of containing the disease.

Our contributions regarding the effectiveness of the contact tracing process in terms of the task of identifying infected nodes, and in terms of the task of finding the source, can be summarized as follows. First, we study how the effectiveness of contact tracing is influenced by the tracing window, i.e., the period for which infected individuals are asked to identify their close contacts. In other words, when people are asked to identify who they came in contact with, should this investigation focus on contacts made before these people got infected, after they got infected, or some time in between? Results indicate that shifting the tracing window has a negligible impact on the effectiveness of contact tracing in either task. Second, we study the impact of using depth-based or breadth-based search when tracing contacts. That is, when selecting the individuals whose close contacts will be traced, should the investigation prioritize those who were recently infected, or those whose infection is further into the past? We quantify the degree to which the former increases the chance of finding the infected nodes, the degree to which the latter increases the chance of identifying the source, and the trade-off between the two. Third, we study the relationship between the contact-tracing budget, i.e., the number of people whose contacts are traced, and the effectiveness of the investigation, measured by either the number of identified infections or the network distance to the source. We show that increasing the budget can significantly improve the former measure, but does not significantly improve the latter beyond a certain point due to the diminishing returns. Fourth, we investigate how the effectiveness of contact tracing is affected by the delay between the introduction of the disease to the network and the start of the tracing process. We find that increasing the delay drops the number of identified infections sharply, but has a relatively small effect on source detection. Fifth, we analyze how changing the characteristics of the disease itself can alter the outcome of contact tracing. As it turns out, a greater transmission rate makes it harder to identify currently infected individuals, while a longer presymptomatic period improves the effectiveness of finding the source. Moreover, a greater $R_0$ makes it easier to identify patient zero, but makes it more difficult to contain the disease since the infected individuals are harder to identify. Finally, we compare three strategies of selecting who to test for the virus: testing people at random, prioritizing close contacts of positive cases, or a mixture of the two. Our results indicate that prioritizing close contacts offers superior efficiency, especially if the process of contact tracing is performed during, and not after, the spread of the disease.

## Results

### Experimental procedure

For a given temporal network, *G* we randomly select one of the nodes as the source node $v^\dagger$ and start spreading the infection from it.

In our experiments, we generate the network structure using either the Barabási–Albert ^14^, Erdős–Rényi ^15^, or Watts–Strogatz^16^ model. We add a temporal structure of contacts to the network using the generative model by Holme^17^. In more detail, for any two nodes with an edge between them, they come into contact every one to seven days according to a truncated power-law distribution with exponent 2.2. To model the infection we use a model by Rusu et al.^13^, which at the moment of writing is the leading model of the COVID-19 disease. Even though it is slightly more complex than simple SIR or SEIR models, we selected it for a more accurate simulation of the disease. See " Materials and Methods" section for the technical details about the network generation and the diffusion model. We let the diffusion spread for *T* rounds, each round corresponding to a single day. Unless stated otherwise, we set $T=28$ days in our simulations, making a single simulation run correspond to four weeks. We selected this value to represent the time necessary to discover the disease and assemble a source detection task force. Since we are only interested in detecting the source of a diffusion that spread successfully, we proceed only if at least $10\%$ of the network got infected. We then perform the contact tracing process.

At the beginning of the contact tracing, the set of detected nodes *D* consists of 10 of the $5\%$ most recently infected nodes (chosen uniformly at random). These nodes represent a small set of infections—those that are initially noticed by the authorities—which form the seed of the contact tracing process. It is worth noting that, in some cases, they might have already recovered. We have a limited budget *b*, which indicates the number of people for whom we can perform the contact tracing. The selection of nodes for which we perform contact tracing is based on the values of the *tracing breadth parameter* $\beta _{tr}\in \{1, \ldots , b\}$. Intuitively, increasing the value of $\beta _{tr}$ makes us focus on the most recently infected nodes, while decreasing the value of $\beta _{tr}$ makes us reach further into the past.

We select $\beta _{tr}$ members of *D* that got infected at the earliest time. For every such node $v^*$ we trace their contacts within $\delta$ days. The choice of the days is determined by the *tracing window offset parameter* $\omega _{tr}\in \{0, \ldots , \delta -1\}$. The contacts of the node $v^*$ are traced on the day $\min (\tau (v^*)+\omega _{tr}, T)$ and six preceding days, where $\tau (v^*)$ is the day when $v^*$ got infected. Hence, the last day when the contacts are traced is the day $\tau (v^*)+\omega _{tr}$, unless it would fall after the day *T* (which we assume to be the last day before we start the contact tracing process), in which case taking the minimum causes the day *T* to be the last day. Intuitively, increasing the value of $\omega _{tr}$ shifts the tracing window towards the future, while decreasing the value of $\omega _{tr}$ shifts the tracing window towards the past. For example, for $\omega _{tr}=6$ we will trace the contact of an infected node on days $\{\tau (v^*), \ldots , \tau (v^*)+6\}$ (i.e., during the week following the infection), while for $\omega _{tr}=0$ we perform tracing on days $\{\tau (v^*)-6, \ldots , \tau (v^*)\}$ (i.e., during the week preceding the infection). The probability that $v^*$ remembers the contact that took place at time *t* is equal to $P(t)=e^{-0.001(T-t)}$^18^. Intuitively, the probability that a member of the network remembers a given contact drops with the time that passed since that contact following an exponential function. For example, a member of the network perfectly remembers their contacts taking place on the same day (in such a case $t=T$, hence $P(t)=1$). However, the probability diminishes as they are asked about the contacts taking place further into the past (as the value of $T-t$ increases, the value of *P*(*t*) decreases). We test for infection 10 (chosen uniformly at random) of the nodes with which $v^*$ remembered having a contact within the described window (note that the contacts that are not remembered by $v^*$ will not be perceived by the tracing process). Notice that this way the total number of performed tests is at most 10*b*, i.e., the parameter *b* reflects the amount of available resources both in terms of tracing contacts and performing the tests. We assume that the test is able to correctly identify an infection whether it is symptomatic, presymptomatic, or asymptomatic.

After tracing contacts for all $\beta _{tr}$ nodes we add the newly identified nodes that were infected at some point in time to *D*. We repeat the process until we run out of budget. The pseudocode of the experimental procedure is presented in Section S1 of the Supplementary Materials. Unless stated otherwise, in our simulations we consider the parameter values $\delta =7$ and $\omega _{tr}=3$.

**Figure 1:**
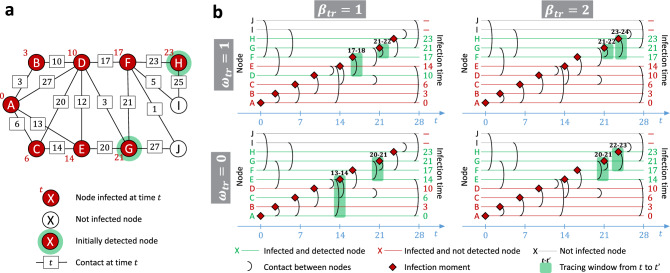
Examples of the tracing process for different values of tracing breadth $\beta _{tr}$ and tracing window offset $\omega _{tr}$. Panel (**a**) presents a temporal network at the end of the infection process started from the node *A*. Red numbers next to the nodes represent infection times, white nodes did not get infected. Numbers on the edges represent contact times. At the beginning of the tracing process only the nodes with green rim are detected. Panel (**b**) presents the results of the tracing process for varying $\beta _{tr}$ and $\omega _{tr}$, with tracing budget $b=2$, and tracing window size $\delta =2$. In every plot horizontal lines represent nodes, with red lines corresponding to infected nodes that did not get detected, green lines to detected infected nodes, and black lines to nodes that did not get infected. Black arcs between lines indicate contacts, with time of the contact represented by the position on the x-axis of the plot. Red rhombuses indicate infection times of the nodes. Green rectangles indicate windows in which contacts of different nodes got traced for the given values of $\beta _{tr}$ and $\omega _{tr}$.

**Figure 2:**
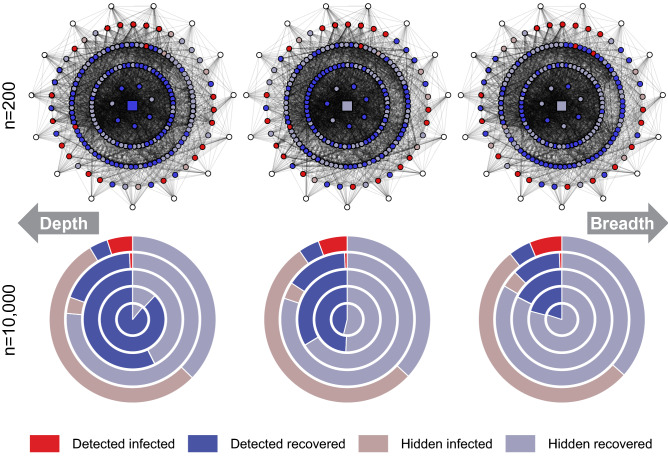
Tracing process for different values of the breadth parameter $\beta _{tr}$ in Watts–Strogatz networks. The first row presents an example of a single simulation of a tracing process in a network with 200 nodes and a budget of $b=10$. The columns correspond to $\beta _{tr}=1$, $\beta _{tr}=5$, and $\beta _{tr}=10$. The source is represented as the square node in the center. The four inner rings of nodes contain nodes that got infected in the first, second, third, and fourth week, with rings further from the middle corresponding to infections later. Red nodes are infected after 28 days. Blue nodes are recovered. A vivid color indicates that the node got detected by the tracing process for the given value of $\beta _{tr}$, and a muted color indicates that the node never got detected (see the legend of the figure). The outer ring contains nodes that never got infected (marked white). The second row presents results for networks with 10,000 nodes and tracing budget $b=100$, aggregated over 1000 simulation runs. The middle circle represents the diffusion source, while the colored rings represent nodes in the first, second, third, and fourth week, with rings further from the middle corresponding to infections later. The columns correspond to $\beta _{tr}=1$, $\beta _{tr}=30$, and $\beta _{tr}=100$.

Figure 1 presents examples of tracing process for different values of $\beta _{tr}$ and $\omega _{tr}$. The infection process starts from the node *A*, which is the source (see how the infection time of node *A* is 0 in Fig. 1a). As a result of the diffusion, all nodes in the network other than the node *I* and the node *J* get infected (see how *I* and *J* are the only white nodes in Fig. 1a). At the beginning of the tracing process, the set of detected nodes consists only of the nodes *G* and *H* (see how they are the only nodes with a green rim in Fig. 1a). If $\beta _{tr}=2$ then the entire tracing budget $b=2$ is used to trace contacts of the set of initially detected nodes (see how in the column for $\beta _{tr}=2$ only the contacts of *G* and *H* are traced in Fig. 1b). On the other hand, if $\beta _{tr}=1$ then first only the contacts of *G* are traced (as it was infected earlier than *H*). As a result, we obtain information about new infected nodes (i.e., node *F* for $\omega _{tr}=1$, or nodes *F* and *E* for $\omega _{tr}=0$). We then trace the contacts of the earliest detected infected nodes (i.e., node *F* for $\omega _{tr}=1$, or node *E* for $\omega _{tr}=0$). As for the $\omega _{tr}$ parameter, notice how for $\omega _{tr}=1$ the contacts are traced at the day of the infection and the subsequent day, while for $\omega _{tr}=0$ they are traced on the day of the infection and the preceding day (see the green numbers over green rectangles in Fig. 1b). Finally, notice that changing the values of $\beta _{tr}$ and $\omega _{tr}$ can significantly change the outcome of the tracing process, e.g., the actual source of the infection, node *A*, is detected only for $\beta _{tr}=1$ and $\omega _{tr}=0$ (see how the line corresponding to *A* is green only for this combination of the parameters in Fig. 1b).

### Simulation results

We first evaluate how the parameters $\beta _{tr}$ and $\omega _{tr}$ affect the performance of the tracing process. First, let us see what are the effects of adjusting the breadth parameter $\beta _{tr}$.

The first row of Fig. 2 presents examples of the tracing process for varying values of $\beta _{tr}$ in a small network with 200 nodes, while the second row presents average results for large networks with 10,000 nodes. As can be seen from the figure, focusing on the breadth of the process results in detecting a greater number of currently infected nodes but a smaller chance of identifying the source of infection, while focusing on the depth of the process makes it easier to identify the source, but decreases the number of currently infected nodes we discover.

**Figure 3:**
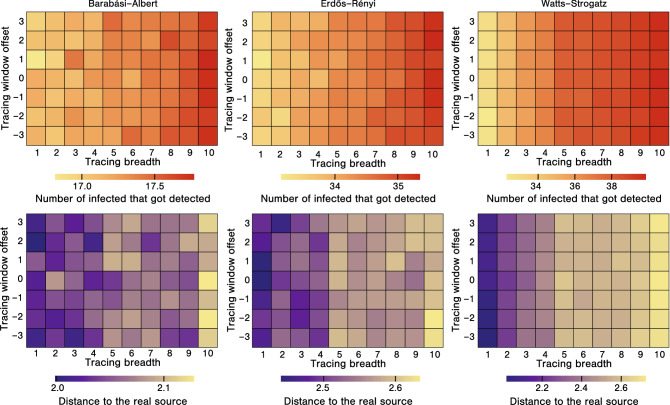
The effectiveness of tracing for varying $\beta _{tr}$ and $\omega _{tr}$. In each plot, the x-axis corresponds to the tracing breadth parameter $\beta _{tr}$ (with greater values indicating more focus on the breadth). The y-axis corresponds to the tracing window offset parameter $\omega _{tr}$ (with greater values indicating the window shifted to the future). The plots in the first row present the number of infected detected by the tracing process, colours closer to red indicate more effective detection. The plots in the second row present the number of edges between the earliest detected infection and the actual source. The colour closer to blue indicates more effective detection. Each column shows results for networks with 10,000 nodes generated using different models, either Barabási–Albert, Erdős–Rényi, or Watts–Strogatz, with tracing budget $b=10$. The results are presented as an average of over 1000 simulations, with a new network generated for every simulation.

Let us now more broadly investigate the effects of changing tracing parameters. Figure 3 presents the results of the simulations with varying values of $\beta _{tr}$ and $\omega _{tr}$. As can be seen from the plots, greater values of the tracing breadth parameter $\beta _{tr}$ result in more effective detection of the currently infected nodes (see colors closer to red in the first row of the figure). On the other hand, smaller values of $\beta _{tr}$ result in closer identifying the source of the infection (see colors closer to blue in the second row of the figure). These findings confirm our observations based on simpler experiments, the results of which were presented in Fig. 2. The value of the $\omega _{tr}$ parameter seems to have little effect on the outcome of the tracing process, i.e., it seems that whether we investigate contacts of the infected person before or after the infection does not make much difference in terms of detection effectiveness. It might be caused by the fact that contacts between a pair of nodes occur regularly in most cases. Hence, even if we do not notice the contact over which the infection spread, we might observe a different contact between the same two nodes. In Fig. 3 we focus on the outcomes of the contact tracing process. To get more insight into the course of the infection itself, see Fig. S2 in the Supplementary Materials, which presents the infection curves from our experiments. In the main article, we focus our attention on experiments with large, randomly generated networks. Nevertheless, to verify our results, we also run simulations on real-life temporal networks. The results of these experiments can be found in Section S2 of the Supplementary Materials. Broadly speaking, the trends observed for real-life networks are the same as those described above for large, randomly generated networks.

**Figure 4:**
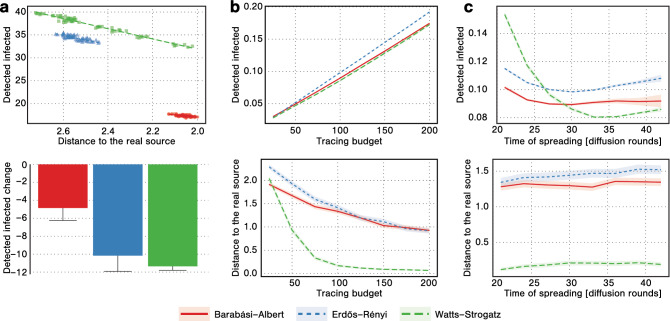
(**a**) Tracing parametrization. The scatter plot presents the number of detected infections and the distance to the real source in settings with varying $\beta _{tr}$ and $\omega _{tr}$ parameters (each point is an average value for a specific combination of $\beta _{tr}$ and $\omega _{tr}$). The lines represent the best fit. The bar plot presents the slopes of the lines, i.e., how much we lose in terms of the number of detected infections when getting one step closer to the source. (**b**) Changing tracing budget. In each plot, the x-axis corresponds to the tracing budget *b*, while the y-axis corresponds to either the number of detected infected nodes, or the number of edges between the earliest detected infection and the real source. (**c**) Changing infection time. In each plot, the x-axis corresponds to the infection spreading time *T*, while the y-axes are the same as in subfigure (**b**). The results in all subfigures are presented as an average of over 1000 simulations for networks with 10,000 nodes, with a new network generated for every simulation using either Barabási–Albert, Erdős–Rényi, or Watts–Strogatz model. The tracing budget is $b=10$ in subfigure (**a**) and $b=100$ in subfigure (**c**). Error bars and colored areas represent $95\%$ confidence intervals.

Our analysis indicates the existence of a trade-off between identifying the source of the infection process and detecting the nodes that got infected. Figure 4a further investigates this compromise. As it can be seen, for a network with 10,000 nodes getting one step closer to identifying the source (in terms of network distance) costs between 4 and 12 identified infections, depending on the structure of the network, with the cost being the greatest in Watts–Strogatz networks, and the smallest in the Barabási–Albert networks. It is worth noting that the total number of detected nodes varies between 15 in Barabási–Albert networks to 40 in Watts–Strogatz networks. Hence the cost of getting one step closer to the source can constitute about one-quarter of all detected infections.

Figure 4b,c present the results concerning the effects of increasing tracing budget and infection spreading time. In Fig. 4b we vary the budget *b* available to the party performing the contact tracing process. Our results suggest that increasing the budget gives a significant advantage in detecting more infected nodes. However, when it comes to identifying the source, the process quickly reaches the state of diminishing returns, where increasing the budget does not provide much better performance. On the other hand, in Fig. 4c we vary the time *T* that the infection process is allowed to run before the contact tracing efforts begin. As can be seen, the percentage of detected infected nodes drops significantly when the infection is allowed to run for a long time. Moreover, identifying the source is getting slightly more difficult as the infection time goes on, although the increase is sublinear with changing *T*.

When it comes to the effectiveness of identifying the source, it might be interesting to compare the considered contact tracing approach to existing dedicated source detection algorithms. In particular, we consider a class of source detection algorithms based on centrality measures, namely Betweenness^2^, Closeness^2^, Degree^2^, Eigenvector^2^, and Rumor^3^ algorithms. As these algorithms work based on the structure of the network, and the set of known infected nodes, we supply them with the information gathered by the contact tracing process. In particular, the network consists of the connections that got successfully traced (i.e., the contacts remembered by the nodes for which we performed the tracing), while the set of known infected nodes is the set of detected infections, *D*. Figure S3 in the Supplementary Materials presents the results of our simulations. As can be seen, the direct use of information gathered during the contact tracing process is consistently more effective in identifying the source than feeding them into existing source detection algorithms. The reason might be the fact that these algorithms were designed to process a different type of information—they expect the entire structure of the network and the complete set of infected nodes, while in reality, the available information is necessarily incomplete.

**Figure 5:**
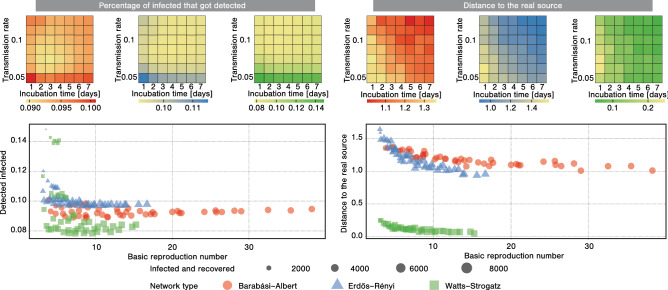
The effectiveness of tracing when changing infection characteristics. In each heatmap, the x-axis corresponds to the presymptomatic period $\mu _p$ of the infection expressed in days, while the y-axis corresponds to the transmission rate $\alpha$ expressed as a probability. In each scatter plot, the x-axis corresponds to the basic reproduction number computed after a week of the infection, while the y-axis corresponds to either the percentage of infected nodes that got detected or the distance to the real source. Each point in a given plot corresponds to a different parameterization of the diffusion process (same as in the heatmaps), with the size of each point corresponding to the number of people that ever got infected. The results are presented for networks with 10,000 nodes generated using different models, either Barabási–Albert, Erdős–Rényi, or Watts–Strogatz, with tracing budget $b=100$, tracing breadth $\beta _{tr}=10$. The results are presented as an average of over 1000 simulations, with a new network generated for every simulation.

Figure 5 presents the results of simulations with changing characteristics of the infection process. More precisely, we consider the infection model described in the "Materials and Methods" section with varying presymptomatic period $\mu _p$ and transmission rate $\alpha$ (all other model parameters remain the same). As can be seen, the increasing transmission rate makes it significantly more challenging to identify currently infected nodes. Similarly, decreasing the presymptomatic period deteriorates the effectiveness of identifying the source. A value that allows encapsulating the severity of the infection is the basic reproduction number $R_0$, indicating the average number of people infected by a single spreader. As evident from both the percentage of identified infections and the distance to the real source drop with the basic reproduction number, indicating that more severe disease strains are more difficult to contain by identifying the infected members of the population but give more hope of discovering the patient zero. Let us now briefly comment on the general trends in how characteristics of the infection (either the presymptomatic period and the transmission rate, or the basic reproduction number) affect the tracing process. For a more virulent, faster-spreading infection it is easier to identify the source, likely due to the fact that the average distance between the source and a given node is much shorter. Intuitively, if any contact with an infected individual resulted in becoming infected, the infection would spread from the source to all nodes along the shortest temporal paths. Similarly, if a larger percentage of the nodes becomes infected, a limited budget available to the party running the contact tracing process is not enough to thoroughly identify all infections. We also perform an analysis of how the outcome of the contact tracing process is affected by the infectious period of the disease. The results of our simulations are presented in Fig. S4 in the Supplementary Materials. When it comes to detecting infections, the situation becomes the most dire for infectious periods between 3 and 4 days (depending on the network model under consideration), as the percentage of detected infections is then minimal. Interestingly, increasing the infectious period makes it easier to identify the source, as it is seemingly more straightforward to backtrack a quickly progressing infection.

**Figure 6:**
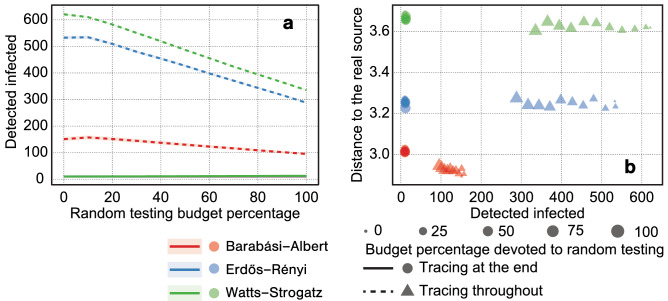
The effectiveness of tracing when ran during the infection process. Panel a presents the number of detected infected nodes based on the percentage of the budget devoted to random testing. Panel b presents the number of detected infected nodes and the distance to the real source in settings with varying percentages of the budget devoted to random testing (represented by point size). The results are presented as an average of over 1000 simulations for networks with 100,000 nodes, with a new network generated for every simulation using either Barabási–Albert, Erdős–Rényi, or Watts–Strogatz model, with tracing budget $b=100$ in each round. Colored areas represent $95\%$ confidence intervals.

Figure 6 presents the results of our experiments with a slightly modified experimental setting. Instead of performing the entire tracing process at a single moment after *T* rounds, we allow the diffusion to continue for another *T* rounds. During each of these rounds, we assume a budget of $b=100$. We spend a percentage *p* of this budget on testing randomly selected nodes, and the remaining $1-p$ percentage of the budget on testing traced contacts as before. We do not test a node if it has been tested in the last 7 rounds. As a baseline, we consider a case in which the same budget is spent in its entirety at the end of the 2*T* rounds period. As can be seen from the figure, we detect a greater number of currently infected nodes when we devote a smaller part of the budget to random testing. It suggests that focusing on contact tracing rather than random testing can not only bring us closer to finding the source of infection, but also help us identify people that are infected at the moment. Moreover, performing contact tracing throughout the process is much more effective than doing it entirely at the end of the considered period.

## Discussion

In this work, we explored using contact tracing to identify the source of a diffusion process, focusing on a diffusion model of the COVID-19 disease as the subject of our investigation. We found that the effectiveness of contact tracing is only marginally affected by shifting the tracing window, i.e., changing the exact days for which we trace contacts. In contrast, the choice between depth-based and breadth-based search has a significant impact on the effectiveness of contact tracing. The former is beneficial when identifying the source of diffusion, and the latter allows for finding more currently-infected nodes. We also showed that investing more resources into contact tracing can dramatically improve the number of discovered infections but has diminishing returns in terms of source detection. Moreover, we found that letting the diffusion spread for a longer time before starting contact tracing slightly reduces the chances of finding the source but has a highly detrimental effect on identifying infected nodes. We also investigated how the contact tracing results are affected by changing characteristics of the spreading infection process, modeling different potential strains of the disease. While it is easier to pinpoint the source of disease with a greater basic reproduction number, the total number of identified infected individuals is smaller for such strains. Finally, we analyzed the outcomes of mixing contact tracing and random testing during the diffusion process, with our results indicating that the former offers superior performance. Altogether, our analysis reveals that the effectiveness of contact tracing in identifying infections and finding the source follow very different trends, and, in many cases, we can observe a direct trade-off between these two objectives.

Our work is related to a growing body of literature on source detection in social networks. Typical source detection algorithms allow inferring which node was the source of the diffusion process based on the structure of the social network and the information about which nodes are infected^2–5^. Typically these algorithms allow to trace back the origins of diffusion starting from a single node, although there also exist techniques that can detect multiple sources^19^. Other source detection techniques are specifically designed to find the source of diffusion in tree networks^20–22^, or analyze the readings of sensors placed throughout the network before the diffusion takes place^23–25^. However, all of these source detection solutions require information that may not be readily available when tracing the origins of infectious diseases, most notably the structure of the underlying network. The technique based on contact tracing considered in this work overcomes this difficulty by reconstructing the connections between nodes during the tracing process without the need for any preexisting knowledge about the network structure.

The main policy implication of our work comes from the observed trade-off between the effectiveness of contact tracing in terms of finding the source and identifying the infected nodes. Our simulations indicate that, while it is possible to conduct both tasks simultaneously, a performance increase in one of them comes at the cost of a performance decrease in the other. Hence, if the primary goal of the contact tracing process is to curb the spreading of a disease, the emphasis should be on the breadth of the search rather than depth. Such an approach would allow us to maximize the effectiveness of containing the infection while, at the same time, letting us slowly work towards the secondary goal of identifying patient zero. Our results suggest that another key factor is the promptness with which the contact tracing process is started. This finding indicates the need to implement procedures to initiate the process as soon as the first signs of a major infection event are detected. Altogether, a promptly initiated and properly guided contact tracing process can be a crucial tool for combating the next global pandemic.

## Materials and methods

### Temporal networks

Let $\langle T \rangle$ denote a time interval of *T* discrete time steps, i.e., $\langle T \rangle = \{0, \ldots , T-1\}$. We will sometimes refer to a particular $t \in \langle T \rangle$ as the *moment* *t*. We denote by $G = (V,K,T) \in \mathbb {G}$ a temporal network, where *V* is the set of *n* nodes, $K\subseteq V \times V \times \langle T \rangle$ is the set of contacts, and *T* is the duration of the time interval during which the contacts in $K$ take place. We denote a *contact* between nodes *v* and *w* at moment *t* by (*v*, *w*, *t*). In this work we only consider *undirected* temporal networks, i.e., we do not discern between contacts (*v*, *w*, *t*) and (*w*, *v*, *t*). Moreover, we assume that networks do not contain self-contacts, i.e., $\forall _{v \in V} \forall _{t \in \langle T \rangle }(v,v,t) \notin K$. We denote all contacts of a given node *v* at time *t* by $K_G(v,t)$. To make the notation more readable, we will often omit the temporal network itself from the notation whenever it is clear from the context, e.g., by writing $K(v,t)$ instead of $K_G(v,t)$.

We consider the following network generation models:

- *Barabási–Albert* model^14^—model generating preferential-attachment networks.
- *Erdős–Rényi* model^15^—model generating uniform random networks.
- *Watts–Strogatz* model^16^—model generating small-world networks. In our simulations we set the rewiring probability to 0.25.

After generating the structure of the network connections we add contact times to them using the generative model for contact sequences by Holme^17^, with $k_{min} = 1$, $k_{max} = 7$, $\gamma = 2.2$, and $\mu = 0.9$.

### Spreading model

We use a spreading model of the COVID-19 disease by Rusu et al.^13^ In this model, every node in the network is in one of the following states: susceptible *S*, exposed but not infectious *E*; infectious presymptomatic $I_p$, infectious asymptomatic $I_a$, infectious symptomatic $I_s$, hospitalized *H*, recovered *R*, or dead *D*. The probabilities of transitions between states in a given round are presented in Table 1, while the descriptions of the model parameters and the values used in our simulations are summarized in Table 2.

**Table 1:** Transition probabilities for the diffusion model.

Transition	Probability
$S \rightarrow E$	$\alpha (K^{I_s} + K^{I_p} + K^{I_a})$
$E \rightarrow I_p$	$\epsilon$
$I_p \rightarrow I_a$	$\mu _p p_a$
$I_p \rightarrow I_s$	$\mu _p (1-p_a)$
$I_a \rightarrow R$	$\gamma$
$I_s \rightarrow R$	$\gamma (1-p_H)$
$I_s \rightarrow H$	$\gamma p_H$
$H \rightarrow R$	$\lambda _{H-R}$
$H \rightarrow D$	$\lambda _{H-D}$

**Table 2:** The spreading model parameters summary.

Parameter	Value	Description
$\alpha$	.0791	Transmission rate
$K^X$	$\in \mathbb {N}$	Number of contacts in state *X*
$\epsilon ^{-1}$	3.7	Latency period
$\mu _p^{-1}$	1.5	Presymptomatic period
$p_a$	.5	Probability of being asymptomatic
$\gamma ^{-1}$	2.3	Infectious period
$p_H$	.1	Probability of being hospitalized
$\lambda _{H-R}$	.083	Recovery rate
$\lambda _{H-D}$	.0031	Death rate

## Supplementary Information

Supplementary Information.

## Data Availability

The datasets generated and/or analysed during the current study are available in an online repository, https://figshare.com/articles/dataset/Contact_tracing_data/21790001.

## Code availability

The code allowing to recreate the results of the current study is available online as a Java Eclipse project, https://github.com/mjwaniek/ContactTracing.
