# Author Correction: Trading contact tracing efficiency for finding patient zero

**DOI:** 10.1038/s41598-023-28809-4

**Published:** 2023-01-27

**Authors:** Marcin Waniek, Petter Holme, Katayoun Farrahi, Rémi Emonet, Manuel Cebrian, Talal Rahwan

**Affiliations:** 1 Computer Science, Science Division, New York University Abu Dhabi, Abu Dhabi, UAE; 2 Aalto University, Espoo, Finland; 3 Kobe University, Kobe, Japan; 4 University of Southampton, Southampton, UK; 5 CNRSLaboratoire Hubert Curien UMR 5516, UJM-Saint-Etienne, Université de Lyon, Saint‑Étienne, France; 6 Center for Humans and Machines, Max Planck Institute for Human Development, Berlin, Germany; 7 Statistics Department, Universidad Carlos III de Madrid, Madrid, Spain; 8 UC3M-Santander Big Data Institute, Universidad Carlos III de Madrid, Madrid, Spain

Correction to: *Scientific Reports* 10.1038/s41598-022-26892-7, published online 30 December 2022

The original version of this Article contained an error in Affiliation 1, which was incorrectly given as ‘New York University Abu Dhabi, Abu Dhabi, UAE’.

The correct affiliation is listed below:

Computer Science, Science Division, New York University Abu Dhabi, Abu Dhabi, UAE.

The original Article and accompanying Supplementary Information file have been corrected.

