# Supplementary material for: Trading contact tracing efficiency for finding patient zero
Source: Sci Rep. 2022 Dec 30;12:22582. doi: 10.1038/s41598-022-26892-7 (PMC9801158; doi:10.1038/s41598-022-26892-7)
Supplement: Supplementary file 1 — Supplementary Information. [file 41598_2022_26892_MOESM1_ESM.pdf]

Supplementary materials for

Trading contact tracing efficiency for finding patient zero

Marcin Waniek[a], Petter Holme[b,c], Katayoun Farrahi[d], Rémi Emonet[e], Manuel Cebrian[f,g,h,*], and Talal Rahwan[a,*]

[a]Computer Science, Science Division, New York University Abu Dhabi, Abu Dhabi, UAE
[b]Aalto University, Espoo, Finland
[c]Kobe University, Kobe, Japan
[d]University of Southampton, Southampton, UK
[e]Univ Lyon, UJM-Saint-Etienne, CNRS, Laboratoire Hubert Curien UMR 5516, Saint-Etienne, France
[f]Center for Humans and Machines, Max Planck Institute for Human Development, Berlin, Germany
[g]Statistics Department, Universidad Carlos III de Madrid, Madrid, Spain
[h]UC3M-Santander Big Data Institute, Universidad Carlos III de Madrid, Madrid, Spain
[*]Joint corresponding authors: manuel.cebrian@uc3m.es, talal.rahwan@nyu.edu

This document is structured as follows:

- **Section S1** (*page 2*) presents the details of the experimental procedure;
- **Section S2** (*page 3*) presents the results of experiments with real-life networks;
- **Section S3** (*page 5*) presents the supplementary figures.

S1 Details of the Experimental Procedure

The detailed pseudocode of the experimental procedure is presented as Algorithm 1. In the loop in lines from 1 to 4, we generate the diffusion process with source $v^\dagger$ that infects at least 10% of the network nodes. In line 5 we select the initially infected nodes that are discovered by the party running the tracing process, and that will be the starting points of tracing. In lines from 6 to 9 we initialize the variables used during the tracing process, which takes place in the loop in lines from 10 to 23, as long as we do not run out of the budget b^*. In a single execution of the loop, we will trace contacts of β_{tr} nodes, and newly detected infections will be collected using variable D^* initialized in line 11. Tracing contacts of a given node takes place in a single execution of the loop in lines from 12 to 22. In line 14 we select the node v^*, the contacts of which will be traced. We add v^* to the set of traced nodes in line 15, and we compute the last day of the tracing window t_0 in line 16 (we will trace the contacts of v^* on day t_0 and δ preceding days). In line 17 we identify the contacts of v^* within the tracing window that v^* remembers. Out of these, we randomly select 10, and we test them for infection in the loop in lines from 18 to 21. More precisely, in line 19 we add w (a contact of v^*) to the set of tested nodes H, and if it is infected (which is checked in line 20), we record this fact in line 21. After tracing the contacts of β_{tr} nodes, the newly detected infections are added to the set of known infections D in line 23. Only now can we trace the contacts of nodes in D^*. The results of the tracing process are returned in line 24.

Algorithm 1 The experimental procedure.

Input: Temporal networks $G = (V, K, T)$, diffusion model M, tracing budget b, tracing breadth parameter β_{tr}, tracing window offset parameter ω_{tr}, tracing window size δ.

Output: The set of infected nodes I, the set of recovered nodes R, and the set of detected nodes D

1: **repeat**
2: $v^\dagger \leftarrow$ select uniformly at random from V ▷ the source of infection
3: $I, R, \tau \leftarrow M(v^\dagger, t)$ ▷ the set of infected nodes, the set of recovered nodes, and the infection times
4: **until** $|I| \geq |V|/10$
5: $D_0 \leftarrow$ uniformly at random select 10 of the 5% most recently infected nodes
6: $D \leftarrow D_0$ ▷ The set of detected nodes
7: $H \leftarrow D_0$ ▷ The set of tested nodes
8: $F \leftarrow \emptyset$ ▷ The set of traced nodes
9: $b^* \leftarrow b$ ▷ The remaining budget
10: **while** $b^* > 0$ **do**
11: $D^* \leftarrow \emptyset$
12: **for** $i \leftarrow 1, \ldots, \beta_{tr}$ **do**
13: **if** $b^* > 0$ **then**
14: $v^* \leftarrow \arg\min_{v \in D \setminus F} \tau(v)$ ▷ Node the contacts of which will be traced
15: $F \leftarrow F \cup \{v^*\}$
16: $t_0 \leftarrow \min(\tau(v^*) + \omega_{tr}, T)$ ▷ The last day of the tracing window
17: $C \leftarrow \{w \in K_G(v^*) : \exists_{(v^*,w,t) \in K} t_0 - \delta < t \leq t_0 \wedge$ coin toss with probability $e^{-0.001(T-t)}\}$
18: **for** $w \in$ randomly select 10 from $C \setminus H_{\beta_{tr},\omega_{tr}}$ **do**
19: $H \leftarrow H \cup \{w\}$
20: **if** $w \in I \cup R$ **then**
21: $D^* \leftarrow D^* \cup \{w\}$
22: $b^* \leftarrow b^* - 1$
23: $D \leftarrow D \cup D^*$
24: **return** I, R, D

S2 Experiments with Real-Life Networks

In this section, we present the results of our experiments with real-life temporal networks. In particular, we consider the following datasets:

- **Hospital** [6]—a network of contacts between patients and health care workers in a geriatric unit of a university hospital collected using wearable proximity sensors, consisting of 73 nodes and $1,381$ edges;
- **Dormitory** [2]—a network of contacts of students living in a dormitory collected via Bluetooth, consisting of 74 nodes and $2,516$ edges;
- **Office** [1]—a network of face-to-face contacts of staff of an office building collected using RFID badges, consisting of 219 nodes and $16,725$ edges;
- **Primary school** [5]—a network of contacts of students and teachers in a primary school collected via radio frequency identification devices consisting of 238 nodes and $5,541$ edges;
- **Conference** [4]—a network of face-to-face contacts between attendees of a medical conference collected using RFID badges, consisting of 403 nodes and $65,355$ edges;
- **Copenhagen** [3]—a network of contacts of university students collected via Bluetooth as part of the *Copenhagen Networks Study*, consisting of 672 nodes and $21,318$ edges.

For each of these networks we perform the same experimental procedure as for the random networks in the main article.

The results of our simulations are presented in Figure S1. As can be seen, the results are largely consistent with those presented for large random networks in Figure 3 in the main article. In particular, adjusting the tracing breadth parameter β_{tr} seems to be more impactful than the tracing window offset parameter ω_{tr}, with greater values of β_{tr} resulting in identifying more infections, but being further away from detecting the source.

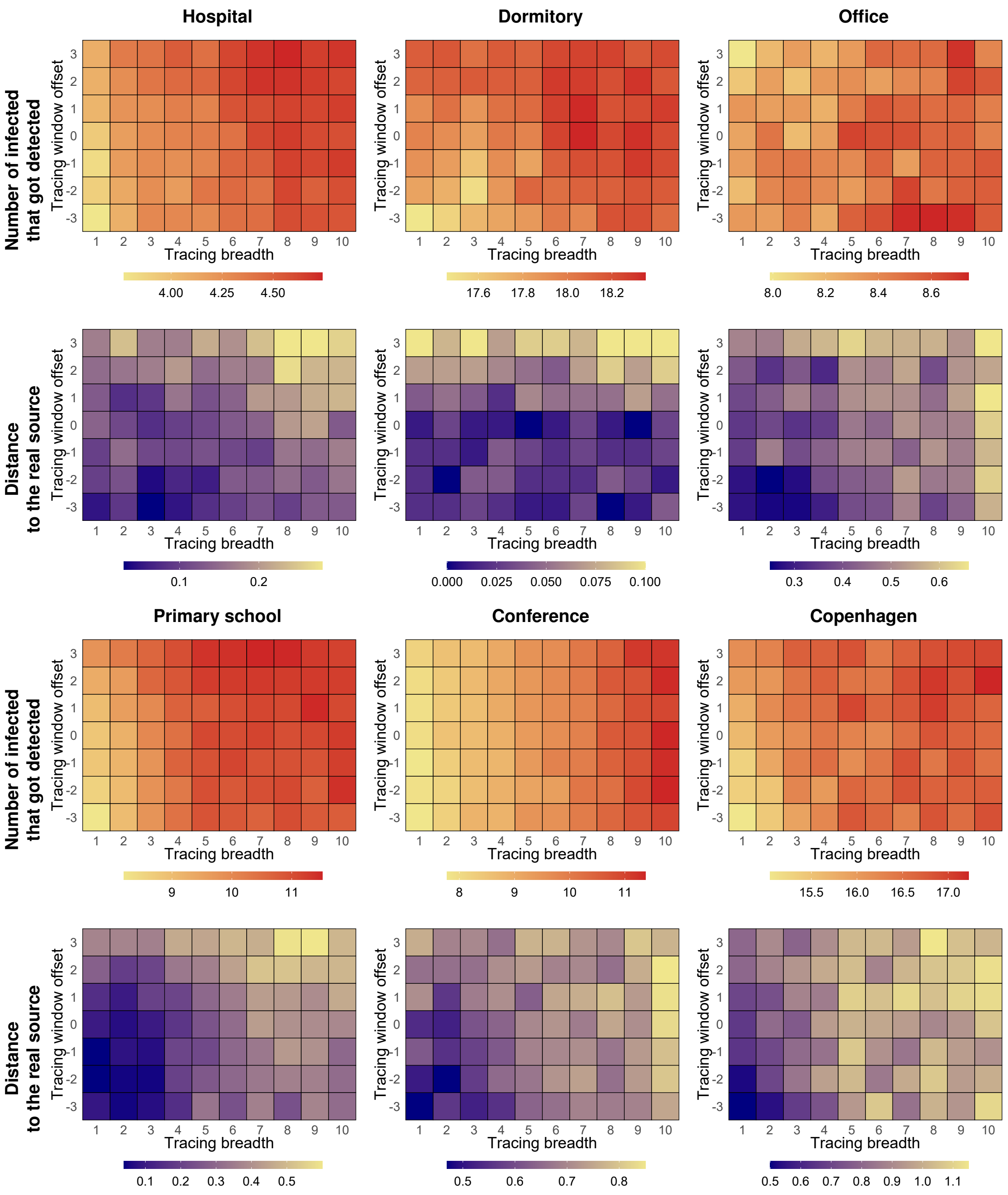

Figure S1: **The effectiveness of tracing for varying β_{tr} and ω_{tr} in real-life networks.** In each plot, the x-axis corresponds to the tracing breadth parameter β_{tr} (with greater values indicating more focus on the breadth). The y-axis corresponds to the tracing window offset parameter ω_{tr} (with greater values indicating the window shifted to the future). The plots in the first and third row present the number of infected detected by the tracing process, colours closer to red indicate more effective detection. The plots in the second and fourth row present the number of edges between the earliest detected infection and the actual source. The colour closer to blue indicates more effective detection. Each pair of plots shows results for different real-life network, with tracing budget $b = 10$. The results are presented as an average of over 100 simulations, with a new infection process generated for every simulation.

S3 Supplementary Figures

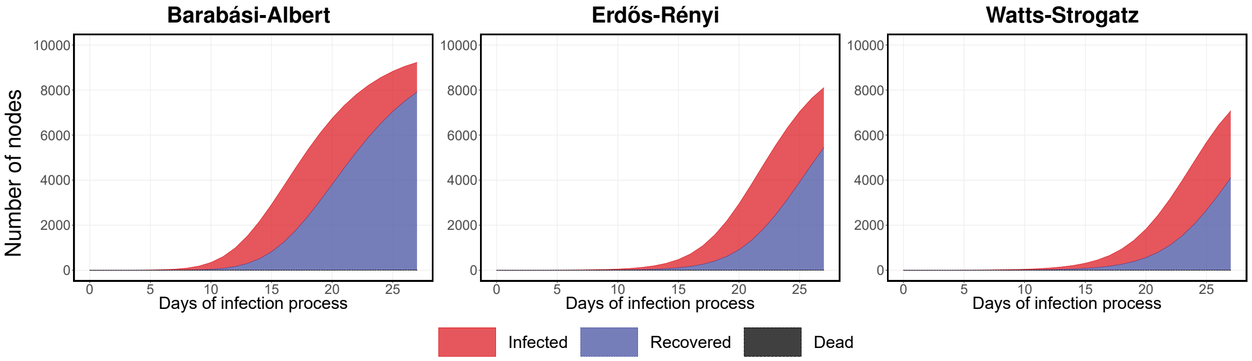

Figure S2: **The infection curves in our experiments.** In each plot, the x-axis corresponds to the time of the infection process expressed in days, while the y-axis corresponds to the number of nodes in a given state. The results are presented for networks with 10,000 nodes generated using different models, either Barabási-Albert, Erdős-Rényi, or Watts-Strogatz. The results are presented as an average of over 1,000 simulations, with a new network generated for every simulation.

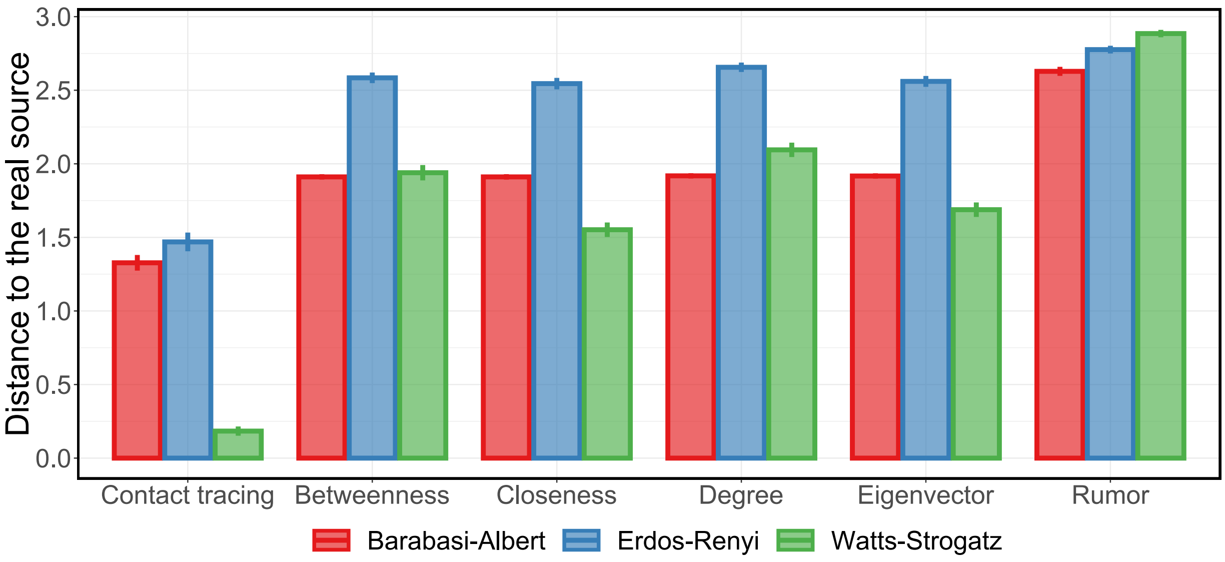

Figure S3: **The comparison of the effectiveness of source detection while contact tracing to other source detection algorithms.** In the plot, each group of bars corresponds to different method of source detection, while the y-axis corresponds to the distance from the detected source to the real source. The results are presented for networks with $10,000$ nodes generated using different models, either Barabási-Albert, Erdős-Rényi, or Watts-Strogatz, with tracing budget $b = 100$, and tracing breadth $\beta_{tr} = 10$. The results are presented as an average of over $1,000$ simulations, with a new network generated for every simulation. The error bars correspond to the 95% confidence intervals.

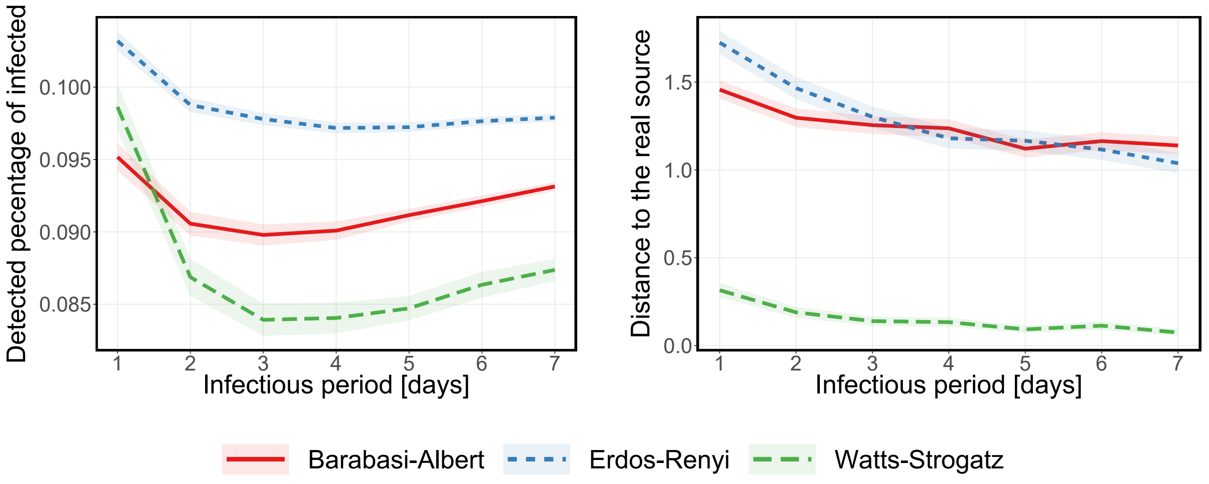

Figure S4: **The effectiveness of tracing when changing infectious period.** In each plot, the x-axis corresponds to the infectious period γ^{-1} of the infection expressed in days, while the y-axis corresponds to either the percentage of infected nodes that got detected or the distance to the real source. The results are presented for networks with $10,000$ nodes generated using different models, either Barabási-Albert, Erdős-Rényi, or Watts-Strogatz, with tracing budget $b = 100$, tracing breadth $\beta_{tr} = 10$. The results are presented as an average of over $1,000$ simulations, with a new network generated for every simulation. The colored areas correspond to the 95% confidence intervals.

References

[1] M. Genois, C. L. Vestergaard, J. Fournet, A. Panisson, I. Bonmarin, and A. Barrat. Data on face-to-face contacts in an office building suggest a low-cost vaccination strategy based on community linkers. *Network Science*, 3:326–347, 9 2015.

[2] A. Madan, M. Cebrian, S. Moturu, K. Farrahi, and A. Pentland. Sensing the 'health state' of a community. *IEEE Pervasive Comput.*, 11(4):36–45, 2012.

[3] P. Sapiezynski, A. Stopczynski, D. D. Lassen, and S. Lehmann. Interaction data from the copenhagen networks study. *Scientific Data*, 6(1):1–10, 2019.

[4] J. Stehlé, N. Voirin, A. Barrat, C. Cattuto, V. Colizza, L. Isella, C. Régis, J.-F. Pinton, N. Khanafer, W. Van den Broeck, et al. Simulation of an SEIR infectious disease model on the dynamic contact network of conference attendees. *BMC Med.*, 9(1):87, 2011.

[5] J. Stehlé, N. Voirin, A. Barrat, C. Cattuto, L. Isella, J.-F. Pinton, M. Quaggiotto, W. Van den Broeck, C. Régis, B. Lina, et al. High-resolution measurements of face-to-face contact patterns in a primary school. *PloS one*, 6(8):e23176, 2011.

[6] P. Vanhems, A. Barrat, C. Cattuto, J.-F. Pinton, N. Khanafer, C. Régis, B.-a. Kim, B. Comte, and N. Voirin. Estimating potential infection transmission routes in hospital wards using wearable proximity sensors. *PloS one*, 8(9):e73970, 2013.
